# S-Adenosylmethionine Inhibits Cell Growth and Migration of Triple Negative Breast Cancer Cells through Upregulating MiRNA-34c and MiRNA-449a

**DOI:** 10.3390/ijms22010286

**Published:** 2020-12-30

**Authors:** Alessandra Coppola, Concetta Paola Ilisso, Antonietta Stellavato, Chiara Schiraldi, Michele Caraglia, Laura Mosca, Giovanna Cacciapuoti, Marina Porcelli

**Affiliations:** 1 Department of Precision Medicine, University of Campania “Luigi Vanvitelli”, Via L. De Crecchio 7, 80138 Naples, Italy; alessandra.coppola@unicampania.it (A.C.); concettapaola.ilisso@unicampania.it (C.P.I.); michele.caraglia@unicampania.it (M.C.); marina.porcelli@unicampania.it (M.P.); 2 Department of Experimental Medicine, University of Campania “Luigi Vanvitelli”, Via L. De Crecchio 7, 80138 Naples, Italy; antonietta.stellavato@unicampania.it (A.S.); chiara.schiraldi@unicampania.it (C.S.)

**Keywords:** S-Adenosylmethionine, triple negative breast cancer, cancer therapy, microRNA, cell growth and migration, miRNA-34c, miRNA-449a

## Abstract

Triple-negative breast cancer (TNBC) is one of the most common malignancies worldwide and shows maximum invasiveness and a high risk of metastasis. Recently, many natural compounds have been highlighted as a valuable source of new and less toxic drugs to enhance breast cancer therapy. Among them, S-adenosyl-L-methionine (AdoMet) has emerged as a promising anti-cancer agent. MicroRNA (miRNA or miR)-based gene therapy provides an interesting antitumor approach to integrated cancer therapy. In this study, we evaluated AdoMet-induced modulation of miRNA-34c and miRNA-449a expression in MDA-MB-231 and MDA-MB-468 TNBC cells. We demonstrated that AdoMet upregulates miR-34c and miR-449a expression in both cell lines. We found that the combination of AdoMet with miR-34c or miR-449a mimic strongly potentiated the pro-apoptotic effect of the sulfonium compound by a caspase-dependent mechanism. For the first time, by video time-lapse microscopy, we showed that AdoMet inhibited the in vitro migration of MDA-MB-231 and MDA-MB-468 cells and that the combination with miR-34c or miR-449a mimic strengthened the effect of the sulfonium compound through the modulation of β-catenin and Small Mother Against Decapentaplegic (SMAD) signaling pathways. Our results furnished the first evidence that AdoMet exerts its antitumor effects in TNBC cells through upregulating the expression of miR-34c and miR-449a.

## 1. Introduction

Breast cancer is the most commonly diagnosed invasive cancer and the second-leading cause of mortality among women worldwide [1,2]. The incidence rate of breast cancer has increased rapidly over the past few decades and, although treatments have substantially improved, a high percentage of patients succumb to the disease due to the progression and development of metastases [3,4]. Among the breast cancer molecular subtypes, triple-negative breast cancer (TNBC) shows the greatest invasiveness, a higher risk of metastasis, and a worse prognosis [5,6].

Tumor metastasis is responsible for up to 90% of cancer-related deaths so, prevention and treatment of metastasis are key to improving clinical outcomes [7]. Metastasis is a multistep process that includes migration and invasion of cancer cells. Acquisition of invasive traits by tumor cells involves morphological and functional changes associated with epithelial–mesenchymal transition (EMT), a highly regulated multistage trans-differentiation process that requires loss of cell-to-cell junctions, loss of epithelial polarity, and the acquisition of migratory and invasive features of mesenchymal phenotype [8,9,10].

EMT has been implicated in breast cancer progression and metastasis and is also involved in cancer stem cell expansion and chemoresistance to cancer treatment. For this reason, finding a natural molecule without significant toxic side effects and capable of reducing the spread of cancer cell is a major clinical challenge and recently intensive efforts have been focused on understanding the molecular mechanisms, as well as new targets for suppressing these processes [8,9,10]. Many natural compounds have been studied in recent years for their potential in improving the therapeutic efficacy of chemotherapy drugs, bypassing resistance to anticancer drugs or reducing the side-effects of chemotherapy. Among natural compounds, the methyl donor S-adenosyl-L-methionine (AdoMet, also called with the acronym SAM) has emerged as one of the most interesting and well-studied naturally-occurring compounds for its therapeutic properties for several common diseases, including cancer. 

AdoMet, the second most extensively-used enzyme cofactor after ATP, performs a wide of well-documented biological functions in all living cells and it is the linking of three primary metabolic pathways: transmethylation, polyamines biosynthesis, and transsulfuration [11,12]. Given the importance of AdoMet in cellular metabolism, it is not surprising that this molecule is being studied as a possible therapeutic agent for the treatment of various clinical disorders. Indeed, the role and therapeutic potential of AdoMet in the treatment of various human diseases such as depression, liver disease, and osteoarthritis are well known in the literature [13,14,15]. Interestingly, AdoMet is available as a food supplement and its chemopreventive action is selective in targeting cancer cells but not normal cells. In addition, at pharmacological doses AdoMet has an excellent tolerability record without side effects [16,17]. Notably, in the last decade many in vitro and in vivo studies have shown promising anti-cancer properties of AdoMet, and growing scientific interest is now focusing on identifying the biological mechanisms and signal transduction pathways related to the antitumoral activity of this physiological compound [18,19,20,21,22,23,24]. 

Experimental evidence has been reported showing that AdoMet is able to regulate, through an epigenetic mechanism, the expression of genes playing a crucial role in cell migration, invasion, and metastasis [25,26,27,28,29,30,31]. In prostate cancer cells, colorectal cancer cells, and osteosarcoma cells, the methyl donor AdoMet is able to reverse the hypomethylated state of prometastatic genes, including urokinase-type plasminogen activator (uPA) and zinc-dependent matrix metalloproteinases (MMPs), which play a key role in the degradation and remodeling of the extracellular matrix and are also involved in the modulation of all stages of carcinogenesis, from tumor initiation to metastasis [26,27,28]. AdoMet showed a significant inhibitory effect on the migratory and invasive ability of Cal-33 and JHU-SCC-011 cells, two head and neck squamous carcinoma cell lines, through modulation of AKT, β-catenin, and SMAD signaling pathways [29]. The antimetastatic properties of AdoMet have also been demonstrated in several non-invasive and invasive human breast cancers, where the sulfonium compound induced a strong inhibition of tumor cell invasion in vitro and tumor growth and metastasis in vivo [25,30,31]. AdoMet blocked the invasiveness and metastatic properties of highly invasive MDA-MB-231 breast cancer cells by significantly inhibiting the expression of uPA and MMP2 [25]. Furthermore, AdoMet synergized with the DNA methylation inhibitor 5-aza-2-deoxycytidine to suppress uPA expression in vitro and reduce breast tumor volume and lung metastases in vivo [30]. AdoMet in combination with 25-hydroxyvitamin has been shown to reduce the proliferation and clonogenic survival of a panel of breast cancer cell lines in vitro and to inhibit tumor growth, lung metastases, and colonization of breast tumor cells at the skeleton in vivo [31]. 

Recent experimental and clinical studies have improved our knowledge of tumor metastasis formation, a dynamic program triggered by complex regulatory networks involving transcription factors, non-coding RNAs, epigenetic modulators, and exogenous inducers by the tumor microenvironment [8,9,10]. Among non-coding RNAs, microRNAs (miRNA or miR) have emerged, in the last decade, as key players in tumorigenesis, through the post-transcriptional regulation of the main modulators involved in cell cycle progression, apoptosis, autophagy, as well as migration and invasion [32,33,34,35]. Many studies have demonstrated that the aberrant expression of miRNAs plays a causal role in breast cancer progression and directly contributes to cell proliferation and metastasis [33,35,36,37,38]. Due to their fundamental role in tumorigenic and metastatic processes miRNAs have been indicated as key targets in the diagnosis, prognosis and therapy of cancer [32,33,34,35,36,37,38,39,40]. 

Our research group has thoroughly investigated the antiproliferative and proapoptotic role exerted by AdoMet in breast cancer [24,41,42] and recently provided the first evidence that in hormone-positive breast cancer cells AdoMet is able to regulate the expression of miRNA-34a, miRNA-34c, and miRNA-486-5p which play a crucial role in the development and maintenance of the tumor cell phenotype [43]. 

Here, we reported that in MDA-MB-231 and MDA-MB-468 TNBC cells AdoMet upregulated the expression of miR-34c and miR-449a, well-known regulators of specific oncogenes and modulators of tumor growth and cancer metastasis in breast cancer [44,45,46,47]. We also showed that the treatment of cells with AdoMet in combination with either miR-34c or miR-449a mimics significantly enhanced AdoMet-induced apoptosis. Finally, by time lapse and monitoring the main molecular parameters related to migration and EMT we demonstrated that the combined treatments potentiated the ability of AdoMet in inhibiting cancer cell migration, strongly suggesting that in MDA-MB-231 and MDA-MB-468 cells miR-34c and miR-449a acted as important mediators of AdoMet-induced inhibition of cell migration. The obtained findings furnished further evidence for AdoMet as a potential proapoptotic and antimetastatic agent for TNBC treatment.

## 2. Results

### 2.1. AdoMet Up-Regulated miR-34c and miR-449a Expression in MDA-MB-231 and MDA-MB-468 TNBC Cells

Emerging evidence suggests that miRNAs play important roles in the pathogenesis of many types of human cancers by modulating different genes in the context of signaling pathways involved in tumor promotion or suppression [32,33,34,35,36,37,38]. 

MiRNA-34c and miRNA-449a, that belong to miRNA-34/449 superfamily, share several target genes and a very similar seed sequence, a conserved heptameric sequence comprising nucleotides 2–7 at the 5′ end of the miRNA, essential for binding to target mRNA [44,45,46,47]. MiRNA-34c and miRNA-449a are downregulated in a wide range of cancers, including human breast cancer suggesting their function as potential tumor suppressors [43,44,45,46,47]. Notably, the up-regulation of miRNA-34 and miRNA-449 has been demonstrated to regulate oncogenic pathways including cell proliferation, metastases and apoptotic pathways [43,44,45,46,47,48,49,50,51]. 

**Figure 1:**
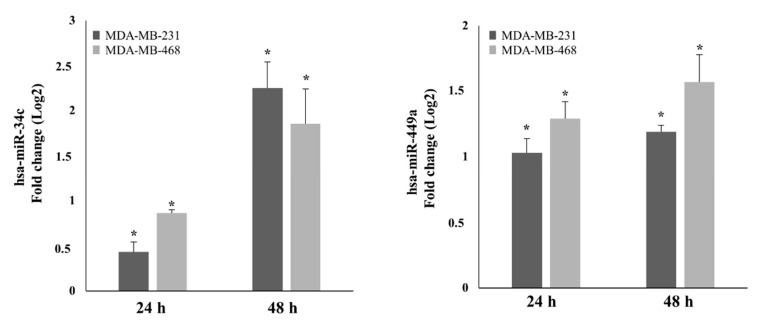
Effect of AdoMet on miR-34c and miR-449a expression in MDA-MB-231 and MDA-MB-468 cells. MDA-MB-231 and MDA-MB-468 cells were treated with 500 μM AdoMet for 24 and 48 h. The relative expression of miR-34c and miR-449a was analyzed by qRT-PCR following normalization with U6 endogenous control. The analysis was carried out by triplicate determination of at least 3 separate experiments. The results are expressed as fold change (Log2) ± SD, * *p* < 0.05.

To obtain insight into the functional mechanism underlying AdoMet’s anticancer effects in TNBC cells, we evaluated its ability to induce the expression of miR-34c and miR-449a by performing quantitative real-time PCR (qRT-PCR) analysis with pre-designed probe-primer sets after 24- and 48 h treatment of MDA-MB-231 and MDA-MB-468 cells with 500 µM AdoMet. The results obtained showed that the relative expression of the two miRNAs was up-regulated by AdoMet in both TNBC cell lines. As shown in Figure 1, after 48 h, miR-34c expression was up-regulated about 2.25-fold and 1.85-fold in MDA-MB-231 and MDA-MB-468 cells, respectively, while the miR-449a level resulted approximately about 1.19-fold and 1.57-fold higher than the corresponding untreated cells. The observed AdoMet-induced up-regulation of miR-34c and miR-449a clearly highlighted the ability of the sulfonium compound to reprogram the expression of non-coding miRNA-34/449 superfamily in TNBC.

### 2.2. Overexpression of miR-34c and miR-449a Enhances the Pro-Apoptotic Effect of AdoMet in MDA-MB-231 and MDA-MB-468 Cells

**Figure 2:**
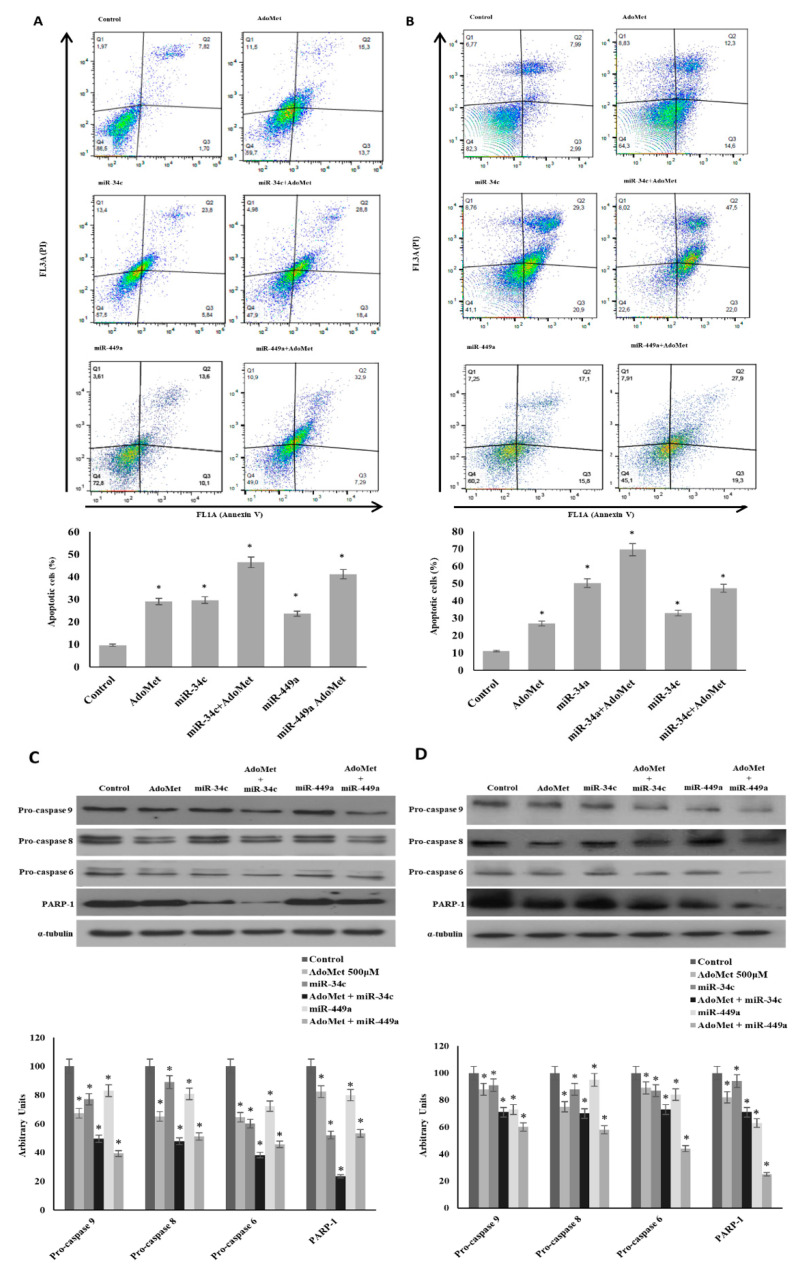
Effect of AdoMet/miR-34c and AdoMet/miR-449 combination on apoptosis and levels of some relevant apoptosis-related proteins in MDA-MB-231 and MDA-MB 468 cells. MDA-MB 231 and MDA-MB 468 cells were transfected with 100 nM miR-34c or miR-449a mimic supplemented or not (Control) with 500 μM AdoMet for 72 h. Apoptosis of MDA-MB 231 (**A**) and MDA-MB 468 cells (**B**) was evaluated by FACS analysis. Representative dot plots of both Annexin V-FITC and propidium iodide (PI)-stained cells are shown. The different quadrants report the percentage of cells: Viable cells, lower left (Q4); early apoptotic cells, bottom right (Q3); late apoptotic cells, top right (Q2); and non-viable necrotic cells, upper left (Q1). For each sample 2 × 10^4^ events were acquired. The analysis was carried out by triplicate determination of at least 3 separate experiments. The lower left and lower right histogram plots show the percentage of apoptotic cells, respectively, for a single treatment. * *p* < 0.05 versus untreated cells (Control). The expression levels of pro-caspase 9, pro-caspase 8, pro-caspase 6, and PARP-1 were detected by Western blot analysis using the total cell lysates of MDA-MB-231 (**C**) and MDA-MB-468 (**D**). The densitometric analysis was reported. Data are reported as percentage of protein expression of untreated control (100%). The house-keeping protein α-tubulin was used as loading control. The images are representative of three immunoblotting analyses obtained from at least three independent experiments. Uncropped images of Western blots are reported in Figure S1.

To explore the antitumor activity of AdoMet in TNBC cells, we first tested the ability of the sulfonium compound to induce apoptosis in MDA-MB-231 and MDA-MB-468 cells. The cells were treated with AdoMet 500 µM and the apoptotic process was evaluated after 72 h by flow cytometry. As shown in Figure 2A,B, treatment with AdoMet resulted in increased accumulation of apoptotic cells in both TNBC cell lines compared to controls accompanied by reduced levels of pro-caspase-8 and -9 which are initiator caspases of the extrinsic and intrinsic apoptotic pathways, respectively, and pro-caspase 6, an executioner caspase (Figure 2C,D). AdoMet-induced activation of caspase cascade was further highlighted by the cleavage of poly (ADP-ribose) polymerase (PARP), a known marker of cells undergoing apoptosis. These findings indicated that AdoMet could effectively induce apoptosis in MDA-MB-231 and MDA-MB-468 cells via activation of caspase signaling pathway. To evaluate whether the AdoMet-induced upregulation of miR-34c and miR-449a was involved in the antiproliferative effect exerted by AdoMet in MDA-MB-231 and MDA-MB-468 cells, we performed transfection experiments with either miR-34c or miR-449a mimics and we evaluated the modulation of apoptotic cell death in AdoMet-treated and untreated cells using flow cytometry-based Annexin V/propidium iodide (PI) assay.

Firstly, by qRT-PCR we demonstrated that, the transfection of cells with miR-34c or miR-449a mimics effectively up-regulates miR-34c and miR-449a transcriptional levels in the two TNBC cell lines reaching a value approximately double (data not shown). Next, to analyze the antitumor activity of miR-34c and miR-449a we assessed apoptosis induction. Our results showed that in both MDA-MB-231 (Figure 2A) and in MDA-MB-468 cells (Figure 2B) the ectopic expression of miR-34c and miR-449a increased the percentage of apoptotic cells respect to the control. Notably, the treatment with AdoMet of MDA-MB-231 and MDA-MB-468 cells transfected with miR-34c or miR-449a mimic significantly improved the pro-apoptotic effect of the sulfonium compound particularly evident in MDA-MB-468 cells where the overexpression of miRNA-34c caused an increase of apoptotic cells from about 27% to 70%. Figure 2 showed that in MDA-MB-231 (Figure 2C) and MDA-MB-468 cells (Figure 2D) the activation of caspase fragmentation and the degradation of PARP-1 analyzed by Western blotting resulted potentiated by the combination of AdoMet with miR-34c or miR-449a further confirming the data obtained by FACS analysis. 

Altogether, these findings indicated that miR-34c and miR-449a played a tumor suppressive role in TNBC cells and that upregulation of miR-34c and miR-449a by AdoMet mediated AdoMet-induced apoptotic cell death.

### 2.3. AdoMet Inhibits TNBC Cell Migration by up Regulation of miR-34c and miR-449a

In order to evaluate the effect of AdoMet on TNBC cell migration, we performed transfection experiments with miR-34c or miR-449a mimics in highly aggressive and invasive mesenchymal-like MDA-MB-231, and in the basal-like MDA-MB-468, characterized by a relatively low invasive phenotype and potential [52] and then we studied the modulation of migration process in AdoMet-treated and untreated cells.

First, we have assessed that in a short time, such as 24 h, after AdoMet and/or miRNAs treatment the apoptotic cell death evaluated by FACS analyses did not interfere with the migration process evaluated at 24 h in both cell lines because it was not significant and reached, as a maximum, a value 10% more than the control (data not shown).

**Figure 3:**
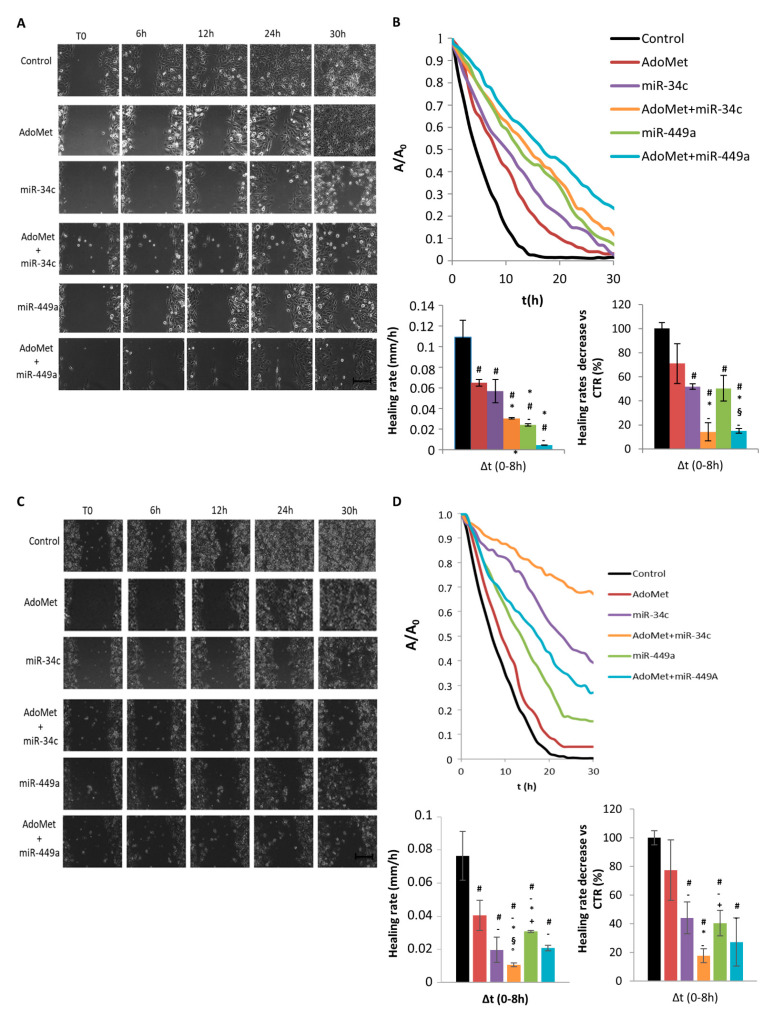
Effect of AdoMet/miR-34c and AdoMet/miR-449a combination on cell migration in MDA-MB-231 and MDA-MB 468 cells. Representative phase-contrast microscopy images showing the wound closure process at five different time points in MDA-MB-231 (**A**) and MDA-MB-468 cells (**C**) transfected with miR-34c or miR-449a and incubated or not (Control) with AdoMet. Images in the panels are relative to a single field of view, taken as qualitatively representative of a given experimental condition. Scale bar, 100 μm. Evolution in time of the wound area A, normalized to the value A_0_ at time 0 (T_0_), for MDA-MB-231 ((**B**) upper) and MDA-MB 468 cells ((**D**) upper) transfected with miR-34c or miR-449a and incubated or not (Control) with AdoMet. Bar diagrams show the values of the healing rate in the time range 0–8 h for control and treated MDA-MB-231 ((**B**) bottom left) and MDA-MB 468 cells ((**D**) bottom left) and the effect of AdoMet and miRNAs on healing rate of MDA-MB-231 ((**B**), bottom right) and MDA-MB-468 ((**D**), bottom right) cells, calculated as percentage of the control in the range time 0–8 h. The data represent an average of three independent experiments; data shown are means ± SD; The statistical significance was analyzed through one-way ANOVA and Tukey post hoc test for comparing a family of 6 estimates: # *p* < 0.05 or less vs. untreated-cells (Control); * *p* < 0.05 or less vs. miR-34c, + *p* < 0.05 or less vs. miR-34c + AdoMet; - *p* < 0.05 or less vs. AdoMet, § *p* < 0.05 or less vs. miR-449a, ° *p* < 0.05 or less vs. miR-449a + AdoMet.

Cell migration was detected in real-time by using video time-lapse microscopy (TLVM), and representative images of the wound closure process are shown in Figure 3A,C for MDA-MB-231 and MDA-MB-468 cells, respectively. We found that, in both cell lines, a qualitatively higher closure of the wound in control respect to AdoMet-treated samples occurred indicating, first of all, that the sulfonium compound inhibited the motility of TNBC cells. When both cell lines were transfected with miR-34c or miR-449a mimics the wounds closed more slowly in AdoMet-treated than in AdoMet-untreated cells demonstrating that the sulfonium compound exerted a synergistic effect with both miRNAs in reducing cell motility over the entire time interval.

**Figure 4:**
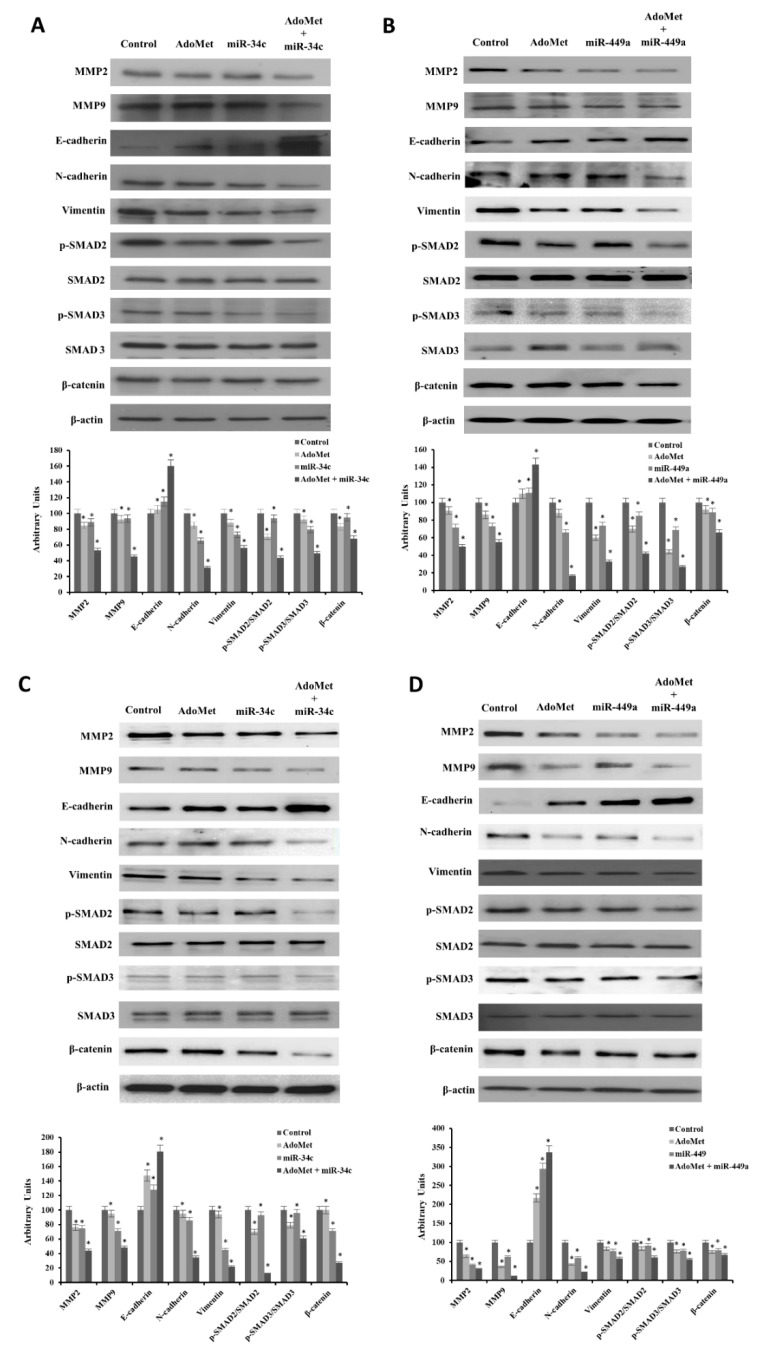
Effect of AdoMet/miR-34c and AdoMet/miR-449 combination on the levels of migration- and EMT-related proteins in MDA-MB-231 and MDA-MB 468 cells. MDA-MB 231 and MDA-MB 468 cells were transfected with 100 nM miR-34c or miR-449a supplemented or not (Control) with 500 μM AdoMet for 48 h. The levels of MMP2, MMP9, Vimentin, *N*-cadherin, E-cadherin, p-SMAD2, SMAD2, p-SMAD3, SMAD3, and β-catenin were detected by Western blot analysis using the total cell lysates of MDA-MB-231 (**A**,**B**) and MDA-MB 468 (**C**,**D**). The densitometric analysis was reported. Data are reported as percentage of protein expression of untreated control (100%). For the equal loading of protein in the lanes, β-actin was used as a standard. The images are representative of three immunoblotting analyses obtained from at least three independent experiments. The intensities of signals were expressed as arbitrary units. * *p* < 0.05 versus untreated cells (Control). Uncropped images of Western blots are reported in Figures S2 and S3.

In Figure 3B and Figure 4D on the top the wound closure dynamics for MDA-MB-231 and MDA-MB-468 cells, respectively, treated with AdoMet and miRNAs, alone and in combination, were analyzed respect to control cells and quantified by plotting A/A0 values as a function of time. The results showed that in both cell lines the control cells closed the wound region faster than the treated cells and that combined AdoMet/miRNAs treatments resulted in more potent inhibition of the wound closure than the single treatment. A further and simpler quantitative measure of the differences among treated and untreated samples was obtained by calculating the wound closure rates in a time interval of 0–8 h. We found that in both cell lines the wound healing rate decreased in treated cells compared to control and more significantly after combined treatments (Figure 3B bottom left and Figure 3D bottom left). In fact, the percentages of wound healing rate respect to the control, measured respectively in MDA-MB-231 and MDA-MB-468 cells, were 14% and 17% in cells treated with AdoMet/miR-34c and 17% and 28% in cells treated with AdoMet/miR-449a (Figure 3B bottom right and Figure 3D bottom right). Altogether, these results confirmed the ability of AdoMet to slow the wound closure and provided further evidence of the synergy between AdoMet and miRNA, demonstrating that the inhibition of MDA-MB-231 and MDA-MB-468 cell migration by AdoMet was mediated by upregulation of miR-34c and miR-449a.

### 2.4. AdoMet Decreased the Levels of the Main Migration- and EMT-Related Markers by Upregulating miR-34c and miR-449a

Cancer metastasis begins with detachment of metastatic cells from the primary tumor, followed by an increase in cellular motility and invasion, proteolysis, and resistance to apoptosis. These four essential steps are correlated and influenced by multi-biochemical events and parameters.

To confirm the results of wound healing experiments and to further investigate the mechanism underlying the AdoMet inhibitory effect on the migration of MDA-MB-231 and MDA-MB-468 cells, we performed transfection experiments with miR-34c or miR-449a mimic, and treated the cells with 500 μM AdoMet for 48 h. By Western blot assay we then analyzed the effect of AdoMet and miRNAs, alone and in combination, on the levels of the main markers and signaling pathways related to cell migration and EMT. First, we assessed the expression of MMPs, proteolytic enzymes that play crucial roles in malignant cell migration [53]. As expected, on the basis of the results of time-lapse experiments, the combination of AdoMet/miR-34c or AdoMet/miR-449a improved the decrease in MMP2 and MMP9 levels induced by single treatments (Figure 4). Migrating tumor cells lose the expression of the epithelial marker E-cadherin, a protein involved in the extensive cell adhesions to neighboring cells and the basement membrane and acquire mesenchymal markers, such as *N*-cadherin and vimentin, a protein overexpressed in most epithelial cancers, whose levels are correlated with tumor migration, invasion, and poor prognosis [9,10]. By Western blot analysis we highlighted a remarkable decrease in *N*-cadherin and vimentin and a concomitant increase in E-cadherin, compared to control, both in MDA-MB-231 (Figure 4A,B) and MDA-MB-468 (Figure 4C,D) cells treated with AdoMet and miR-34c or miR-449a mimic alone, indicating that the reduced cell mobility induced by AdoMet or miRNA mimics depended on the inhibition of EMT process. Notably, we found that the combined treatment strongly potentiated the effect obtained with AdoMet. TGF-β/SMAD and β-catenin, signaling pathways have established roles in the migration and EMT progression of TNBC cells [54,55]. So, we examined the status of SMAD proteins, known to modulate the activity of TGF-β ligands and critically important for the regulation of cell development, growth, and EMT [55]. The results reported in Figure 4, indicated that AdoMet inhibited SMAD2 and SMAD3 phosphorylation and induced a decrease of p-SMAD2/SMAD2 and p-SMAD3/SMAD3 protein ratio. Notably, we found that these effects were much more pronounced after combined treatment with AdoMet and miR-34c or miR-449a mimic. Finally, we evaluated the levels of β-catenin, a multifunctional protein that is involved in cell-to-cell adhesions and represents the most important mediator of the canonical Wnt pathway. In TNBC cells inhibition or silencing of β-catenin markedly down-regulates EMT-related transcriptional factors resulting in reversal of EMT, cell migration, and metastasis [54]. Interestingly, our data exhibited a strong down-regulation of β-catenin in MDA-MB-231 (Figure 4A,B) and MDA-MB-468 (Figure 4C,D) cells treated with AdoMet compared to untreated cells that increased in combination with miR-34c or miR-449a.

Overall, these findings indicated that the combination of AdoMet and miR-34c or miR-449a was more effective than the single agents in inhibiting cell migration and EMT suggesting that the antimigration and antimetastatic properties of AdoMet in TNBC cells could be explained, at least in part, by the AdoMet-induced upregulation of EMT-suppressive miRNA-34c and miRNA-449a and involved modulation of TGF-β and β-catenin signaling pathways.

## 3. Discussion

MiRNAs represent a class of small, endogenous non-coding transcripts from 16 to 29 nucleotides that develop post-transcriptional regulation by mRNA cleavage or translation repression, which depend on the degree of complementarity of miRNA-mRNA [32,33,34].

Several studies have demonstrated the important role of miRNAs in the regulation of all essential cellular bioprocesses, including proliferation, differentiation, apoptosis, stress response, and migration [32,33,34,35,36,37,38,39,40]. MiRNA research has become increasingly attractive as evidence is emerging that miRNAs act as key regulators in the pathogenesis of diseases, including cancer. Since aberrant expression of miRNAs is often involved in neoplastic processes, manipulation of miRNAs expression appears to be an attractive and innovative therapeutic approach. There are two important reasons that make miRNA appropriate for this goal. First, miRNAs, as natural antisense nucleotides, have shown reduced immune response and low toxicity compared to drug molecule-based therapy. Second, considering that each miRNA can regulate even a hundred target genes, pleiotropic effects can be obtained by modulating the aberrant expression of a single miRNA.

The high incidence of TNBC and its poor prognosis have led science to find new, more effective and less toxic therapeutic strategies to reduce the chemoresistance of these tumors. Moreover, several scientific evidences indicated that abnormal expression of miRNAs in TNBCs might affect the outcome of chemotherapy [56,57].

In recent years, an increasing number of studies focused on natural anti-cancer compounds as a valuable source of new and less toxic drugs that may be useful in blocking both tumor growth and metastasis. Among the natural compounds, AdoMet, due to its epigenetic modulating properties has been shown to inhibit the progression and the onset of metastases of several tumors by acting on different signal transduction pathways.

Recent studies reported that in human hepatocellular carcinoma (HCC) aberrant expression of miRNA induces dysregulation of methionine adenosyltransferase (MAT) isoforms responsible for maintaining adequate AdoMet cellular levels and that depletion of AdoMet results in increased HCC cell proliferation, a decrease in AdoMet is due to mutations in MAT1A gene. Yang et al. found that in HCC the up-regulation of miR-664, miR-485-3p, and miR-495 induced a lower MAT1A expression, that correlates with a worse prognosis [58]. Inhibition of the expression of these miRNAs raised the level of MAT1A, proving a potential innovative strategy for the treatment of HCC [58]. Koturbash et al. demonstrated the contribution of miR-22 and miR-29b in the inhibition of MAT1A and MTHFR expression during 2-acetylaminofluorene-induced rat HCC, suggesting that downregulation of these genes may be one of the main driver events that promote liver carcinogenesis [59]. Finally, in HepG2 human hepatoma cell line, up-regulation of miR-21-3b by berberine an isoquinoline alkaloid extracted from many medicinal herbs, reduced the expression of MAT2A and MAT2B resulting in increased intracellular AdoMet levels which acted as a key regulator for hepatoma cell proliferation [60].

A direct correlation between the antiproliferative effect of AdoMet and the regulation of miRNAs expression has been reported in breast cancer where AdoMet was able to modulate miR-34a, miR-34c, and miR-486-5p. Ilisso et al. demonstrated that in MCF-7 cells the combined treatment with AdoMet and miR-34a and/or miR-34c greatly enhanced the pro-apoptotic effect of the sulfonium compound by a caspase-dependent mechanism and that the downregulation of miR-486-5p potentiated the autophagic effect of AdoMet by increasing PTEN expression and by inhibiting AKT signaling [43]. To date, this is the only report highlighting the potential of AdoMet to modulate miRNA-mediated epigenetic events associated with breast cancer.

In the present study, we demonstrate for the first time that AdoMet induced apoptosis and inhibited EMT process and migration of MDA-MB-231 and MDA-MB-468 TNBC cells up-regulating miR-34c and miR-449.

In order to highlight a direct correlation between the anti-cancer properties of AdoMet and the variation of miRNA expression in TNBC the first focus of this study was to examine the ability of AdoMet to modulate miR-34c and miR-449a expression. Results reported showed that AdoMet was able to up-regulate miR-34c and miR-449a in MDA-MB-231 and MDA-MB-468 cells. These data, in agreement with findings previously obtained by our research group [43], suggest that AdoMet is an attractive therapeutic agent able to modulate miRNA expression profile in tumoral context.

It is relevant to mention that the miR-34c and miR-449a families were classified as one miRNA superfamily (miRNA-34/449 superfamily) since they share the same seed sequence and targets [45,46,47].

Recent studies have reported that miRNA-34/449 superfamily is downregulated in a wide range of cancers, including human breast cancer and plays a pivotal role in TNBC initiation, progression and metastasis [45,46,47,48,49,61].

MiR-34 family consists of three members (miR-34a, miR-34b, and miR-34c) transcribed from two different sets of genes located on chromosomes 1 and 11. It has been extensively studied that miR-34 family is involved in the control of cancer growth by targeting different tumor-related genes and signaling pathways [43,45,46,47,48,49,61]. Recently, it was also reported the involvement of miR-34 family in the suppression of breast cancer invasion and metastasis [48,62]. Achari and colleagues established that miR-34c exerts the antitumor activity in breast cancer by several mechanisms including the G2/M cell cycle arrest and the suppression of BCL2 and SIRT1 [48].

MiR-449 family consists of three members (miR-449a, miR-449b, and miR-449c) encoded by a cluster located on chromosome 5q11.2 in the second intron of CDC20B, identified as a susceptibility region in cancer [49]. As for miR-34 family, the miR-449 family exhibits low expression in cancers and acts as a tumor suppressor by inhibiting a wide variety of oncogenes. Several studies have shown the beneficial effects of the upregulation of miR-449a in cancer [49,50,63,64,65,66,67]. Very recently it has been demonstrated that up-regulation of miR-449a suppresses the proliferation as well as the migration and invasion of laryngeal carcinoma cells targeting Notch1 and Notch2 down-regulation and it has been suggested that miR-449a, alone or in combination therapy with Notch inhibitors, could be used as a potential tool to treat metastatic laryngeal carcinoma [51].

Despite numerous data reported in the literature, the function and clinical significance of miR-449a in breast cancer remain not fully understood. It has been reported that miR-499 family plays both oncogenic and tumor suppressive roles in different breast cancer cell lines. It has been shown, indeed, that in MCF-10, MDA-MB-231, T47D, and MDA-MB-453 breast cancer cells a down-regulation of miR-449 family inhibits tumor growth, invasion, and metastasis formation, and promotes apoptosis and differentiation [68,69]. On the contrary, in MDA-MB-231 and MCF-7 cells, the over-expression of miR-449a suppresses cell proliferation, clone formation, migration, invasion, and then metastasis formation both in vitro and in vivo by downregulating the zinc finger protein PLAGL2 [49].

The present study showed that AdoMet exhibited antiproliferative activity in TNBC cells and provided evidence on the underlying mechanism. We demonstrated that in MDA-MB-231 and MDA-MB-468 cells AdoMet triggered apoptosis by activating caspase cascade and PARP cleavage and that these effects were more pronounced in cells transfected with miR-34c or miR-449a. These results confirmed the well-documented proapoptotic effect of AdoMet and are in good agreement with the extensive literature reporting miRNA-34/449 as critical regulators of apoptosis (ApoptomiR), [43,47]. Notably, the data also evidenced the role played by miR-34c and miR-449a as important mediators of the antiproliferative effects exerted by AdoMet in MDA-MB-231 and MDA-MB-468 cells suggesting that AdoMet-induced up-regulation of miRNA-34/449 could offer promising opportunities for the treatment of TNBC.

TNBC is a highly aggressive subtype with a strong proliferative capacity and a high risk of distant and/or nearby metastasis. Acquisition of TNBC invasive properties is driven by the aberrant activation of EMT, a highly regulated, multi-step trans-differentiation process considered responsible for TNBC invasion, metastasis, and resistance to treatment [7,8,9,10].

Many literature reports have highlighted the ability of AdoMet to epigenetically inhibit migration and invasiveness of various tumor cells at in vivo and in vitro levels [24,25,26,27,28,29,30,31].

The novel finding of this study, obtained by qualitatively and quantitatively monitoring the real-time migration of TNBC cells in wound-healing assay by time-lapse microscopy and analyzing the main migration- and EMT-related molecular markers, indicated that AdoMet inhibited cell migration and reversed EMT in MDA-MB-231 and MDA-MB-468 cells and that these effects are mediated by miR-34c and miR-449a.

We found that AdoMet caused a decrease in the migration rate of MDA-MB-231 and MDA-MB-468 cells suggesting the ability of the sulfonium compound to lower the aggressiveness of TNBC cells and to potentially reduce their metastatic power. The observation that AdoMet treatment of cells transfected with miR-34c or miR-449a significantly enhanced the inhibitory effects of the sulfonium compound further confirmed the involvement of both miRNAs in this process.

Overexpression of MMPs has been associated with metastasis formation ad unfavorable outcomes in several malignant tumors [53,70,71]. Interestingly, we showed that AdoMet induced a decrease of MMP-2 and MMP-9 levels confirming the antimigratory potential of the sulfonium compound in TNBC cells.

*N*-cadherin is highly expressed in mesenchymal cells. Its upregulation is considered an important marker of EMT and has been shown to promote tumor cell motility and invasion [9,10,72,73]. On the other hand, in epithelial tumor cells, E-cadherin acts as a tumor suppressor, playing an important role in maintaining the phenotype and polarization of the epithelial cell layers. Loss of E-cadherin is associated with carcinogenesis and invasion [9,10,74,75]. In tumor cells, the reduction of E-cadherin expression and the concomitant increase of *N*-cadherin is associated with an increase in migratory and invasive behavior with high prognostic significance [75,76]. It should also be emphasized that during EMT tumor cells significantly modify the cytoskeletal structure with an increase in the expression of vimentin, critically involved in the adoption of a mesenchymal form and in increased motility, for this reason it is considered the main marker of EMT [77].

Our findings showed that AdoMet induced inhibition of MDA-MB-231 and MDA-MB-468 cell migration paralleled by decreased levels of *N*-cadherin and vimentin and increased levels of E-cadherin indicative of the ability of AdoMet to reverse EMT process in these TNBC cells.

TGF-β/SMAD and β-catenin signaling play well-established roles in migration and EMT progression of TNBC cells. TGF-β is constitutively expressed in metastasizing breast cancer and SMAD-mediated TGF-β signaling represents the most potent inducer of EMT process and metastasis to other tissues during breast cancer progression [54,78]. Recent studies show that in TNBC cells the Wnt/β-catenin axis is particularly overactivated and regulates various properties associated with the tumor, such as migration, stem, anchorage-independent growth, and chemosensitivity [54,78].

In line with these reports, we demonstrated that AdoMet caused a decrease of the levels of β-catenin and SMAD-2/3 and their phosphorylated forms, providing evidence that AdoMet inhibited EMT process in TNBC cells through down-modulating TGF-β/SMAD and β-catenin signaling pathways. Notably, the down-regulation of MMPs observed after AdoMet treatment as well as the AdoMet-induced increased levels of E-cadherin, and decreased levels of *N*-cadherin, vimentin, β-catenin, SMAD-2,3 and their phosphorylated forms were all enhanced by the combined treatment of AdoMet with miR-34c or miR-449a providing convincing evidence that AdoMet exhibited its antitumor activity in MDA-MB-231 and MDA-MB-468 cells through up-regulation of these tumor suppressive miRNAs.

Taken together, our data allowed us to propose the possible mechanism underlying the anticancer effects of AdoMet in TNBC cells in which AdoMet-induced upregulation of miRNA 34c/449a represents an early and crucial event. Subsequently, the downregulation of TGF-β/SMAD and β-catenin signaling by direct or indirect targeting plays a role downstream of miRNAs in regulating apoptosis, EMT, and cell migration as shown by the activation of the miRNA 34c/449a-induced caspase cascade, PARP cleavage, increased expression of the epithelial marker E-cadherin, decreased expression of the mesenchymal markers *N*-cadherin and vimentin, reduced levels of metalloproteinase 2/3 and inhibition of cell migration.

Our results helped to increase knowledge on the anticancer mechanisms exerted by AdoMet and suggested that the sulfonium compound, being able to regulate miR-34c and miR-449a, could represent a suitable pharmacological approach for TNBC treatment.

## 4. Materials and Methods

### 4.1. Reagents

AdoMet (New England Biolabs), dissolved in a 5 mM H_2_SO_4_ and 10% ethanol solution, filtered and stored at 4 °C until use. Annexin V-fluorescein isothiocyanate (Annexin V-FITC) Apoptosis Detection kit (eBioscience, San Diego, CA, USA) was used for apoptosis detection. Monoclonal antibodies (mAbs) to caspase 9 (#9508, dilution: 1:1000), caspase 8 (#9746, dilution: 1:1000), PARP (#9532, dilution: 1:1000), β-actin (#3700, dilution: 1:5000 and 1:2000), α-tubulin (#2125, dilution: 1:1000), SMAD2 (#5339, dilution: 1:1000), phospho-SMAD3 (#9520, dilution: 1:1000), SMAD3 (#9523, dilution: 1:1000), MMP9 (#13667, dilution: 1:1000), vimentin (#5741, dilution: 1:1000), β-catenin (#8480, dilution: 1:1000), *N*-cadherin (#13116, dilution: 1:1000), E-cadherin (#14472, dilution: 1:500 and 1:1000) and polyclonal antibodies (polyAbs) to caspase 6 (#9762, dilution: 1:1000) phospho-SMAD2 (#3104, dilution: 1:1000), MMP2 (#4022, dilution: 1:1000) were purchased from Cell Signaling Technology (Danvers, MA, USA). Horseradish peroxidase (HRP)-conjugated goat anti-mouse and goat anti-rabbit secondary antibodies (ImmunoReagents Inc.; Raleigh, NC, USA), RIPA Buffer (Sigma-Aldrich; St. Louis, MO, USA). MiRNA-34c and miRNA-449a mimics, Lipofectamine 2000, mirVANA PARIS Kit, TaqManMiRNA Reverse Transcription Kit, Megaplex RT Primers, TaqMan Universal PCR Master Mix, Opti-minimal essential medium (Opti-MEM) were obtained from Life Technologies (Carlsbad, CA, USA).

### 4.2. Cell Cultures and Treatments

Triple negative human breast cancer cell lines MDA-MB-468 and MDA-MB-231 (American Type Culture Collection; ATCC, Manassas, VA, USA), were cultured at 37 °C in a 5% CO_2_ humidified atmosphere and grown in Dulbecco’s modified Eagle’s medium (DMEM) with 10% fetal bovine serum (FBS), 2 mM L-glutamine and penicillin-streptomycin 50 U/mL. Cells were seeded in 6-well plates at the density of 1.5 × 10^5^ cells/well to achieve 80% of confluence. After 24 h, the cells were treated with 10% FBS fresh medium containing 500 µM AdoMet for 24, 48, and 72 h. Subsequently, fluctuating cells were recovered from culture medium by centrifugation, whereas adherent cells were collected by trypsinization.

### 4.3. Cell Transfections

MDA-MB-468 and MDA-MB-231 cells, at 80% confluence, were transfected with 100 nM miR-34c or miR-449a mimic, diluted in Opti-MEM medium integrated or not (Control) with 500 μM AdoMet, using Lipofectamine 2000 according to the manufacturer’s protocol. Lipofectamine was also used alone as a negative control. On the basis of the various experimental conditions, the cells were collected and then processed to carry out the appropriate analyzes.

### 4.4. MiRNA Detection by qRT PCR

Total RNA was purified using the PARIS mirVANA kit (Invitrogen, Carlsbad, CA, USA), according to the manufacturer’s protocol. The RNA concentration was determined using a NanoDrop 1000 spectrophotometer (Thermo Fisher Scientific, Waltham, MA, USA). Subsequently, using the TaqMan MiRNA Reverse Transcription Kit, single-stranded cDNA was synthesized from total RNA samples.

The expression of individual miRNAs was determined using pre-designed probe-primer sets from Life Technologies (Thermo Fisher Scientific, USA) by quantitative real-time PCR (qRT-PCR) performed on a ViiA7™ Real-time PCR system (Applied Biosystems, Darmstadt, Germany), according to the manufactures.

To normalize total RNA samples, the small-nuclear-U6 was selected as endogenous control. Relative expression of the transcripts was measured by using ViiA7™Real-Time PCR software (Applied Biosystems, Darmstadt, Germany).

### 4.5. Flow Cytometry Analysis of Apoptosis

Annexin V-FITC was used in conjunction with the vital dye propidium iodide (PI) to analyzed the apoptotic occurrence by distinguishing apoptotic (Annexin V-FITC-positive, PI positive) from necrotic (Annexin V-FITC-negative, PI-positive) cells [79]. MDA-MB 468 and MDA-MB 231 were plated in serum-containing media in 6-well plates at the proper density and the day after, cells were transfected with 100 nM miR-34c or miR-449a mimic, with or without 500 µM AdoMet. After 72 h, cells were detached by incubation with EDTA-trypsin, washed twice with phosphate-buffered saline (PBS), and collected by centrifugation. Then, the cells were resuspended in 200 μL of Binding Buffer 1X and incubated with 5 μL Annexin V-FITC and 10 μL PI (20 μg/mL) for 30 min at room temperature, as recommended by the manufacturers. The detection of viable cells, early apoptotic, late apoptotic, and necrotic cells was performed by BD Accuri™ C6 flow cytometer (Becton Dickinson, San Jose, CA, USA). For each sample, 20,000 events were recorded. Analysis was carried out by triplicate determination on at least three separate experiments.

### 4.6. Time-Lapse Video Microscopy

In order to perform in vitro scratch-wound healing assay to evaluate cell migration, MDA-MB-231 and MDA-MB-468 cells incubated or not (Control) with AdoMet and trasfected with miR-34c or miR-499a were seeded on collagen pre-coated 12-well tissue culture plate. Scratch wounds were obtained mechanically with a sterile pipette tip (Ø = 0.1 mm). Detached cells and debris were washed away with PBS. The multiwell was placed on a cage incubator (Okolab S.r.l. Pozzuoli, Italy). Wound closure was monitored for 48 h by TLVM, based on an inverted optical microscope (Zeiss Axiovert 200, Germany) equipped with a CCD-gray-scale camera (ORCA ER, Hamamatsu Photonics, Hamamatsu City, Japan), a microscope stage incubator (CO_2_, T, and air control) that accommodates different wells, a thermostatic bath (LAUDA, Eco Line RE 204, Okolab S.r.l. Pozzuoli, Italy), and a remotely controlled motor that allows micrometric movements and the repositioning of the stage incubator along x, y and z cyclically in time. All environmental conditions required for cell cultures (37 °C and 5% CO_2_ in air, respectively) are maintained by microscope stage incubator. The instrument was controlled by the custom-tailored software OKO-Vision 4.3, (Okolab S.r.l. Pozzuoli, Italy) consisting of OKO-Vision Time Lapse and OKO-Vision Imaging [80]. At regular intervals, several independent fields in the plates were captured using by a high-resolution (1 h).

The wound closure measurements were calculated by the software as Area t (time t of healing)/Area t_0_ (wound areas just after scratching). Each treatment was performed in duplicate (2 wells), each well was observed with at least five fields of view, which were quantified for the respective scratched area over time (healing). The data generated by the software were analyzed to obtain the reduction of the wound area (in squares micrometers) at each time point. Finally, a wound closure rate (cell migration rate) was calculated by dividing the repaired area (expressed after conversion pixel/squared micrometers as mm/h) by the height of the view field (scratch height, h) and then dividing it by the time of observation, the equation used is the following: [(Area t1 − Area t2)/h/(t2 − t1)] expressed as mm/hour. Moreover, the fields of view selected and used to build up the overall averaged curves all had a similar scratch width, ranging from 0.7 to 0.9 mm, corresponding to a wound area of 16–20 mm^2^. The statistical significance of the experiment was ensured by the possibility to visualize contemporarily several fields of view (up to 30–36 h) of the same sample (depending on the delay time chosen by the operator) in the staged incubator. Furthermore, a single field of view (~10 × 10^5^ μm^2^) represented 5% of the total scratch area (~20 × 10^6^ μm^2^) of each well. Because we captured at least five field-views in three repetitions per well, this ensured us that we analyzed 25–30% of the scratch in each specific well. Triplicates were performed for each scratch assay.

### 4.7. Preparation of Cell Lysates

MDA-MB 468 and MDA-MB-231 cells were transfected with 100 nM miR-34c or miR-449a mimic, treated or not with AdoMet 500 µM and after 48 and 72 h, collected by centrifugation, washed twice with ice-cold PBS, and the pellet was lysed using 100 µL of RIPA buffer. After incubation on ice for 30 min, the samples were centrifuged at 18,000× *g* for 30 min a 4 °C and the supernatant was recovered. Protein concentration was performed by Bradford method as previously reported [81].

### 4.8. Western Blotting Analysis

Western blotting analysis was performed as previously reported [82]. All primary antibodies were used at a dilution of 1:1000, except E-cadherin prepared at a dilution of 1:500, while all secondary antibodies were used at a dilution of 1:5000, except E-cadherin prepared at a dilution of 1:2000. Blots were developed using enhanced chemioluminescence detection reagents ECL (Cyanagen, Bologna, Italic) and exposed to X-ray film. All films were scanned by using Image J software 1.48v (U.S. National Institutes of Health, Bethesda, MD, USA).

### 4.9. Statistical Analysis

Statistical analysis was performed as previously reported [82].

## 5. Conclusions

Overall, our results provided the first evidence that AdoMet exerts its antitumor effects in TNBC cells by regulating miRNA expression and gave new information for a better and deeper understanding of the molecular mechanisms underlying the anticancer properties of this naturally-occurring multifunctional sulfonium compound suggesting the use of AdoMet as an attractive chemopreventive and therapeutic strategy miRNA-mediated in TNBC.

Our study also provided significant contribution to the knowledge of miR-34/449 biological functions furnishing the first evidence that in MDA-MB-231 and DA-MB-468 cells miR-34c and miR-449a act as tumor suppressors and inhibitors of the metastatic potential of cancer cells by targeting TGF-β/SMAD and β-catenin signaling pathways.

Taken together, these data suggested the advantage of using natural compounds with pleiotropic effects, such as AdoMet and miRNAs, which can regulate many target genes achieving more efficient modulation than single-target drugs. These discoveries offer another approach for the scientific community in cancer therapy using only natural compounds, and highlight a new tool to fight cancer giving the possibility to recover health by avoiding typical side-effects, improving prognosis, and increasing the percentage of remission. However, it appears that miRNAs are very suitable in combination with AdoMet in cancer therapy and more data are expected in the coming years to deepen the knowledge of the regulation of non-coding RNAs by AdoMet in order to improve currently applied anticancer therapies.

## Data Availability

Not applicable.

## Supplementary Materials

Supplementary Materials can be found at https://www.mdpi.com/1422-0067/22/1/286/s1.

## Abbreviations

AdoMet	S-adenosyl-L-methionine
TNBC	triple negative breast cancer
miRNA or miR	microRNA
EMT	epithelial–mesenchymal transition
uPa	urokinase-type plasminogen activator
MMPs	matrix metalloproteinases
PARP	poly (ADP-ribose) polymerase
TLVM	video time-lapse microscopy
SMAD	Small Mother Against Decapentaplegic
HCC	human hepatocellular carcinoma
FBS	fetal bovine serum
DMEM	Dulbecco’s modified Eagle’s medium
PI	propidium iodide
PBS	phosphate-buffered saline
qRT-PCR	quantitative real-time PCR
Annexin V-FITC	Annexin V-fluorescein isothiocyanate
