# Supplementary material for: S-Adenosylmethionine Inhibits Cell Growth and Migration of Triple Negative Breast Cancer Cells through Upregulating MiRNA-34c and MiRNA-449a
Source: Int J Mol Sci. 2020 Dec 30;22(1):286. doi: 10.3390/ijms22010286 (PMC7795242; doi:10.3390/ijms22010286)
Supplement: Supplementary file 1 [file ijms-22-00286-s001.pdf]

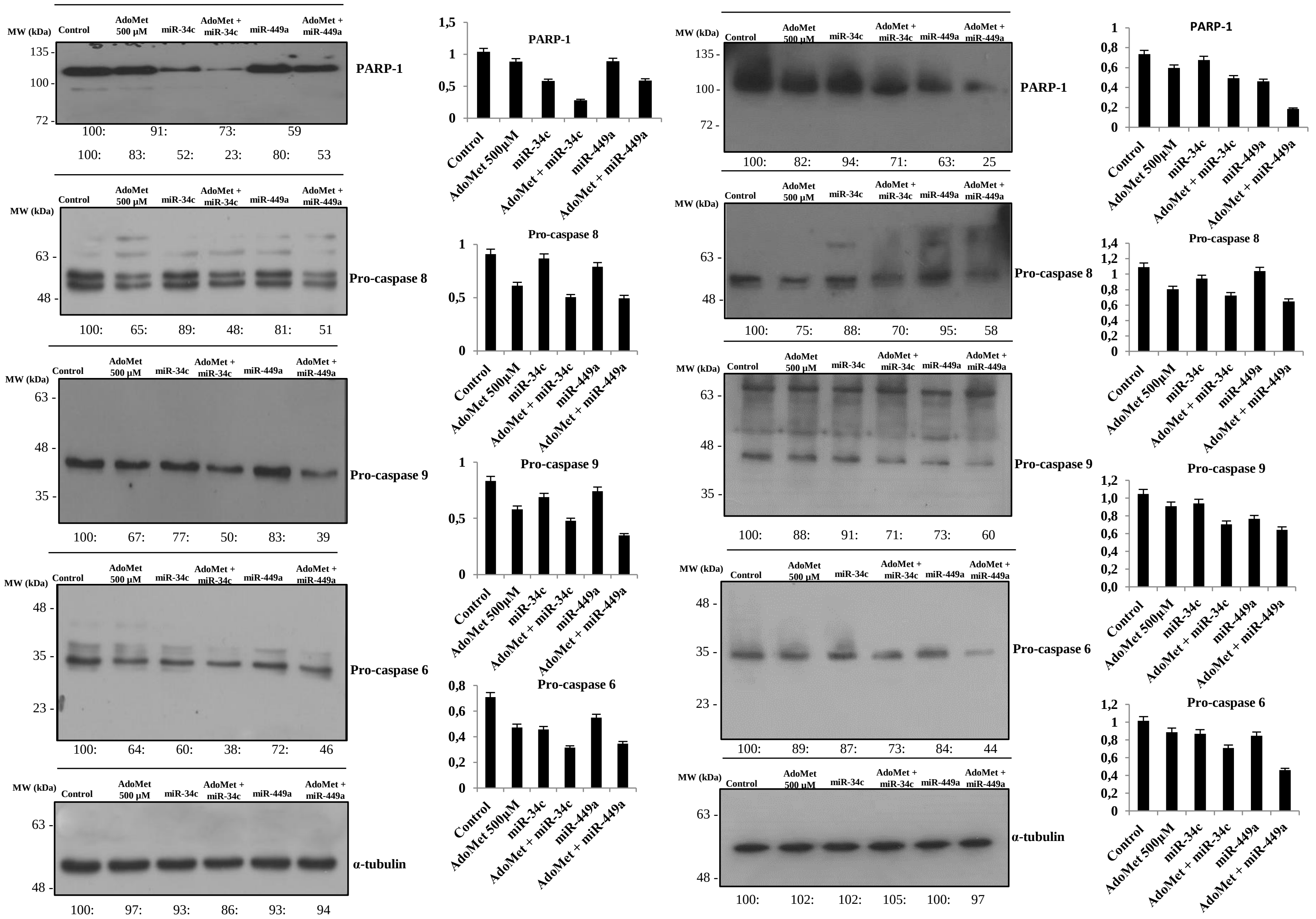

Figure S1: Effect of AdoMet/miR-34c and AdoMet/miR-449 combination on the levels of some relevant apoptosis regulating proteins in MDA-MB-231 and MDA-MB 468 cells. The croppled bloots are used in the main Figure 2.

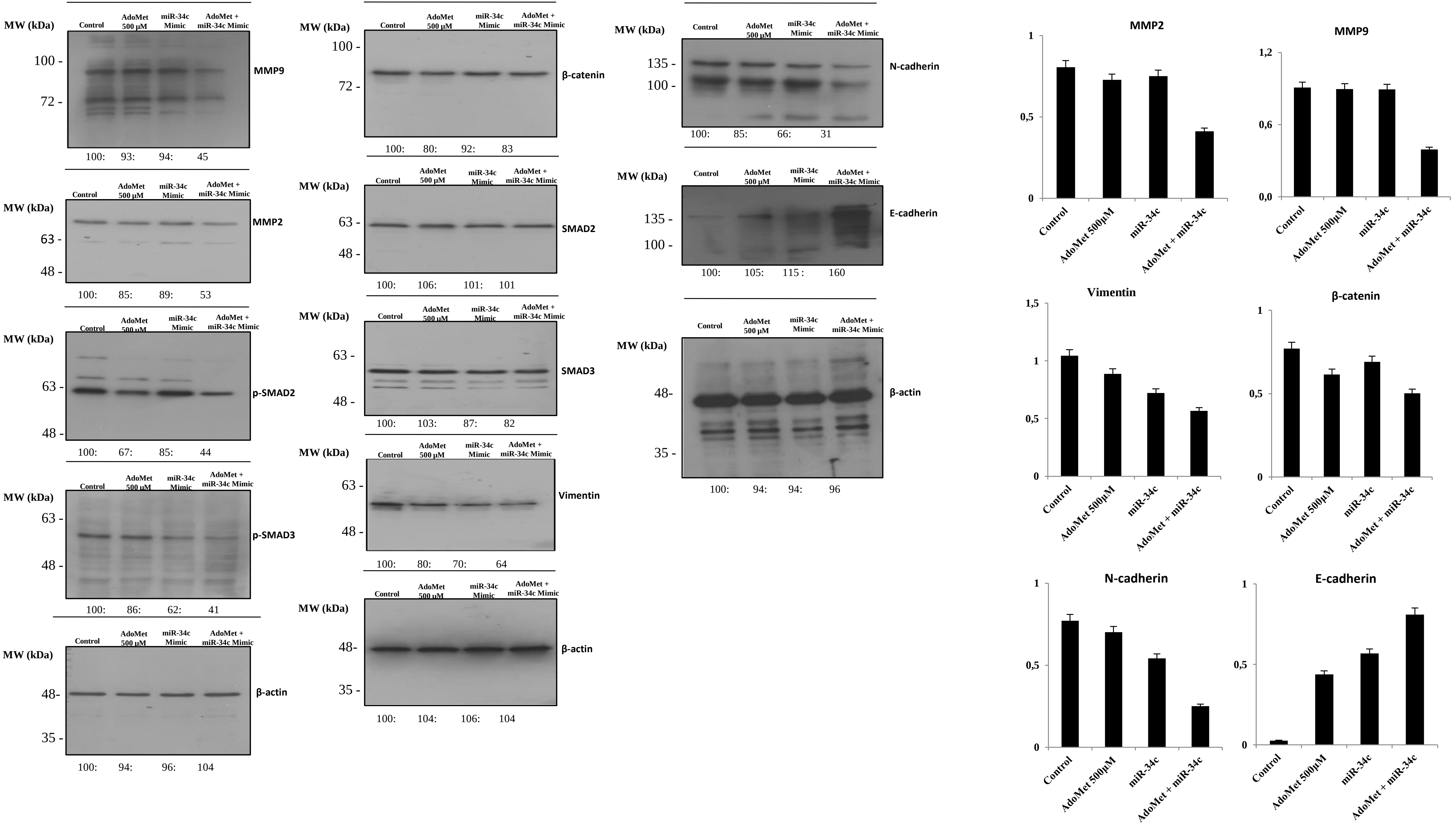
MW (kDa)
Control
AdoMet 500 µM
miR-34c Mimic
AdoMet + miR-34c Mimic
100 -
72 -
MMP9
100: 93: 94: 45
MMP2
63 -
48 -
100: 85: 89: 53
p-SMAD2
100: 67: 85: 44
p-SMAD3
100: 86: 62: 41
β-actin
48-
35 -
100: 94: 96: 104
β-catenin
100: 80: 92: 83
SMAD2
100: 106: 101: 101
SMAD3
100: 103: 87: 82
Vimentin
100: 80: 70: 64
β-actin
100: 104: 106: 104
N-cadherin
135 -
100: 85: 66: 31
E-cadherin
100: 105: 115 : 160
β-actin
100: 94: 94: 96
MMP2
1
0,5
0
MMP9
1,2
0,6
0,0
Vimentin
1,5
1
0,5
0
β-catenin
N-cadherin
E-cadherin
Control
AdoMet 500µM
miR-34c
AdoMet + miR-34c

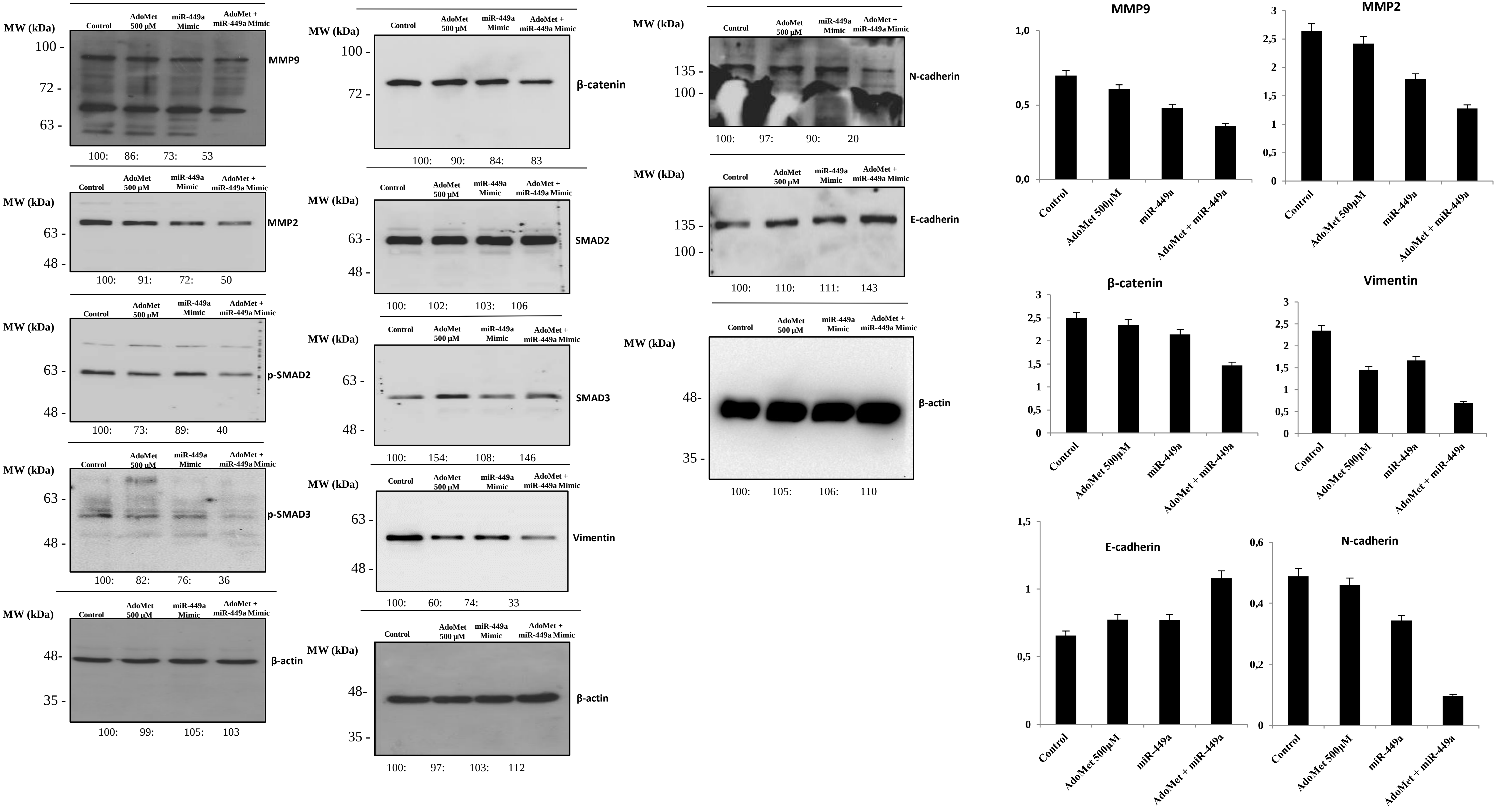

Figure S2: Effect of AdoMet/miR-34c and AdoMet/miR-449 combination on the levels of migration- and EMT-related proteins in MDA-MB-231 cells. The cropped bloots are used in the main Figure (Fig. 4)

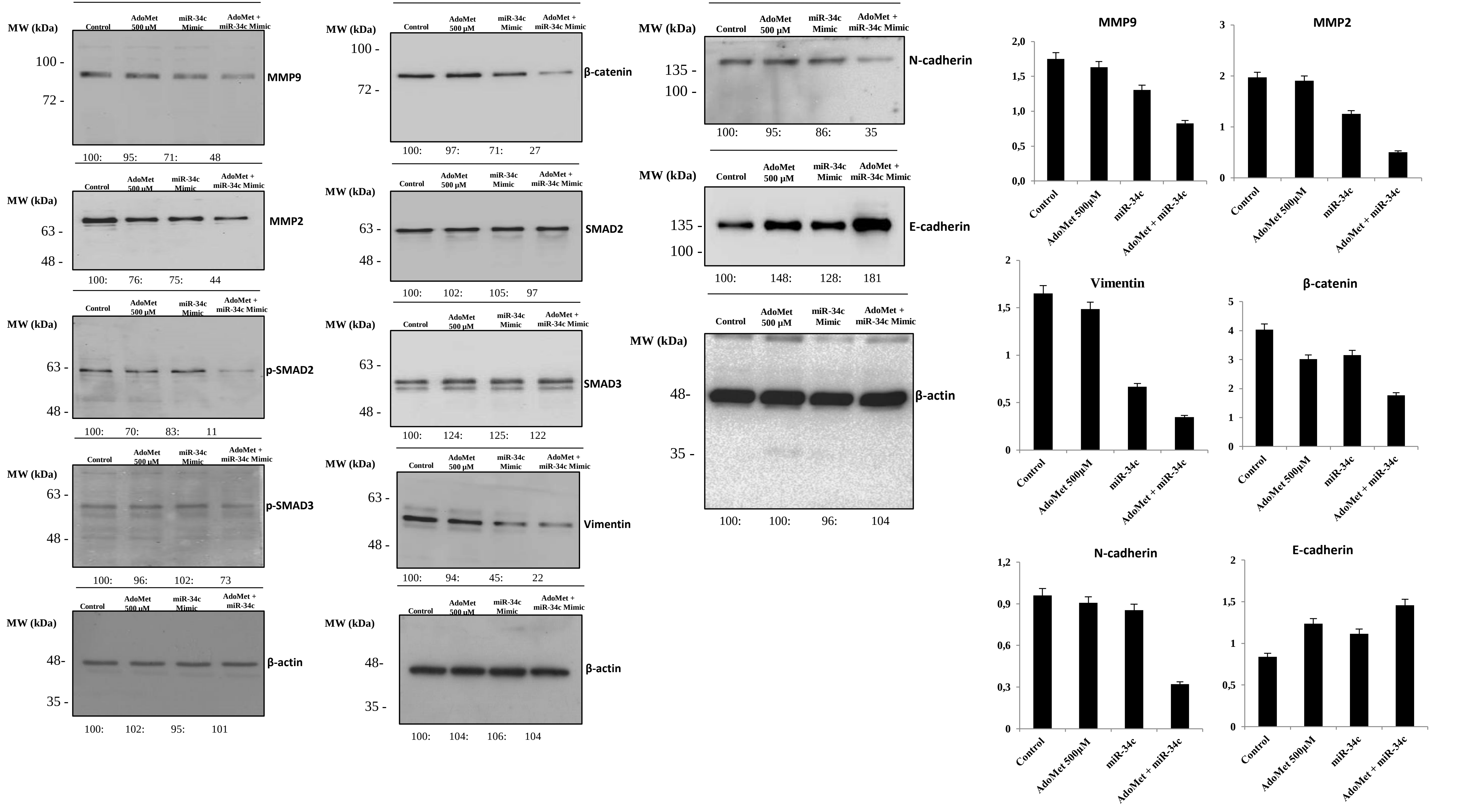
MW (kDa)
Control
AdoMet 500 µM
miR-34c Mimic
AdoMet + miR-34c Mimic
MMP9
100: 95: 71: 48
MMP2
100: 76: 75: 44
p-SMAD2
100: 70: 83: 11
p-SMAD3
100: 96: 102: 73
β-actin
100: 102: 95: 101
β-catenin
100: 97: 71: 27
SMAD2
100: 102: 105: 97
SMAD3
100: 124: 125: 122
Vimentin
100: 94: 45: 22
β-actin
100: 104: 106: 104
N-cadherin
100: 95: 86: 35
E-cadherin
100: 148: 128: 181
β-actin
100: 100: 96: 104
MMP9
MMP2
Vimentin
β-catenin
N-cadherin
E-cadherin
Control
AdoMet 500µM
miR-34c
AdoMet + miR-34c

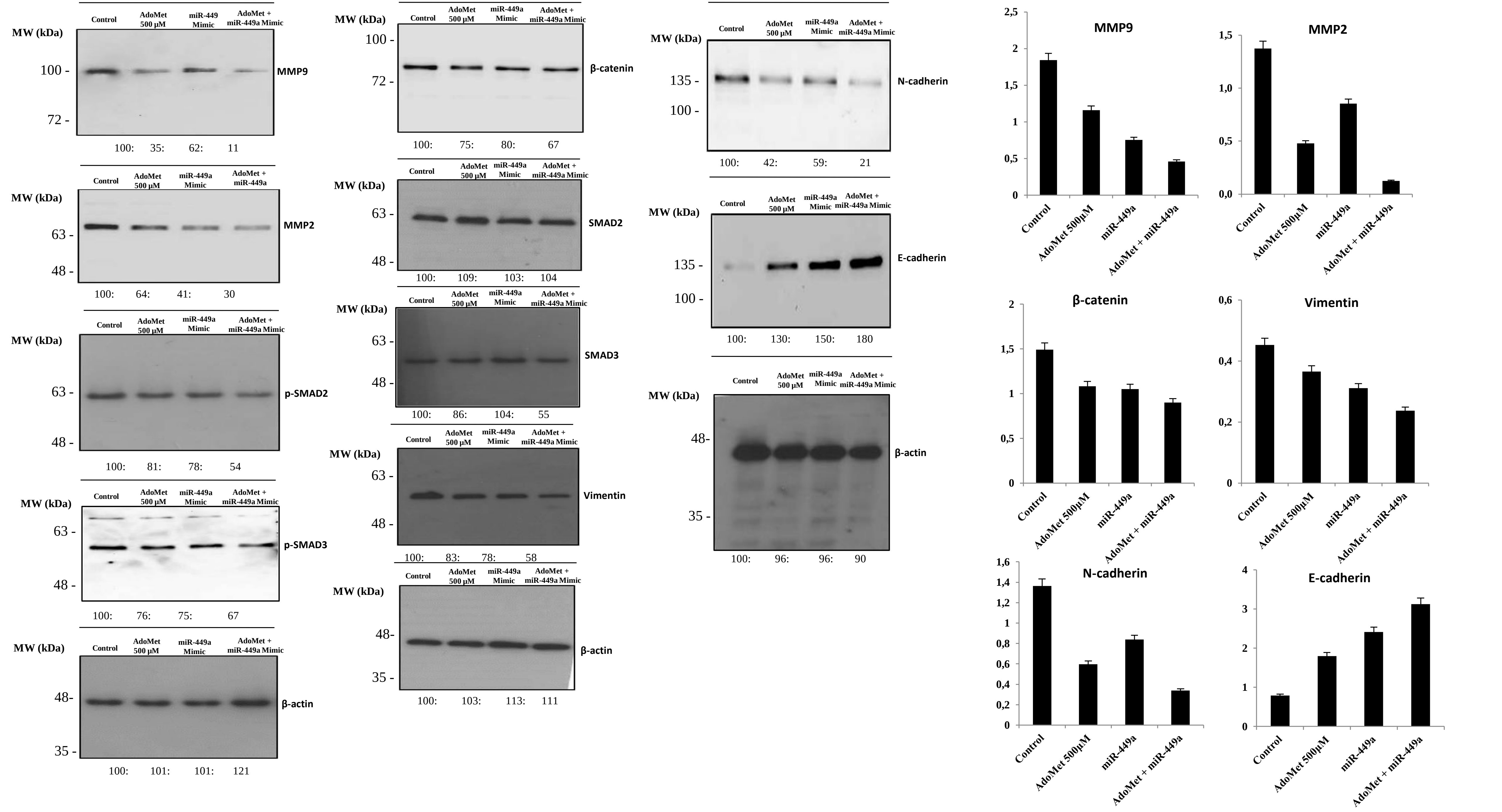

Figure S3: Effect of AdoMet/miR-34c and AdoMet/miR-449 combination on the levels of migration- and EMT-related proteins in MDA-MB-468 cells. The cropped bloots are used in the main Figure (Fig. 4)
